# Carbon Doping in Small Lithium Clusters: Structural, Energetic, and Electronic Properties from Quantum Monte Carlo Calculations

**DOI:** 10.1021/acsomega.4c09963

**Published:** 2025-01-09

**Authors:** Bráulio G. A. Brito, Guo-Qiang Hai, Ladir Cândido

**Affiliations:** † Departamento de Física, Instituto de Ciência Exatas e Naturais e Educação, Universidade Federal do Triângulo Mineiro, Uberaba, Minas Gerais 38064-200, Brazil; ‡ Instituto de Física de São Carlos, Universidade de São Paulo, São Carlos, São Paulo 13560-970, Brazil; § Instituto de Física, Universidade Federal de Goiás, Goiânia, Goiás 74001-970, Brazil

## Abstract

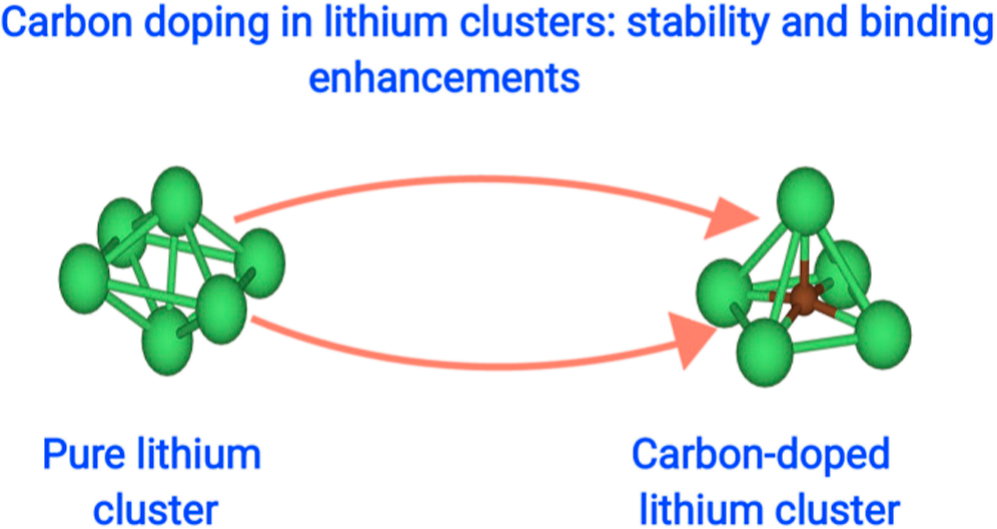

We investigate the energetic and structural properties of small lithium clusters doped with a carbon atom using a combination of computational methods, including density functional theory (DFT), diffusion quantum Monte Carlo (DMC), and the Hartree–Fock (HF) approximation. We calculate the lowest energy structures, total ground-state energies, electron populations, binding energies, and dissociation energies as a function of cluster size. Our results show that carbon doping significantly enhances the stability of lithium clusters, increasing the magnitude of the binding energy by 0.261 ± 0.008 to 1.048 ± 0.003 eV. Carbon substitution also reduces the bond length by approximately 1.00 Å and decreases the coordination number by up to 2.78. The dissociation energy required to remove the doped carbon atom ranges from −7.65 ± 0.02 to −3.33 ± 0.01 eV, which is substantially larger in magnitude than the energy required to remove a lithium atom, varying from −2.81 ± 0.02 to −0.78 ± 0.02 eV. These results indicate that carbon doping enhances cluster stability, as reflected by the increased dissociation energy and changes in bonding characteristics. We compare our findings with available theoretical and experimental data, providing valuable insights into the role of carbon doping in strengthening the stability and bonding properties of lithium clusters.

## Introduction

Doping lithium clusters with carbon leads to unconventional bonding interactions that result in novel structural configurations and electronic behaviors that enhance the overall stability and functionality of these materials.^1,2^ These unique interactions result from the interplay of the electron accepting tendency of carbon and the metallic bonding of lithium, which together enable the formation of clusters with different electronic properties. Such modifications are particularly valuable for improving material performance in the fields of energy storage, catalysis and nanotechnology.^3−7^ In particular, carbon-doped lithium clusters have shown great promise for advancing high-capacity battery technologies and improving the efficiency of catalytic processes.^8,9^

Lithium clusters, whether pure or doped, exhibit remarkable properties that make them particularly noteworthy among metallic clusters. Lithium clusters are known for their intriguing metallic, optical, biological, and even superconducting characteristics.^10−13^ Furthermore, certain lithium clusters display superalkaline properties, allowing them to form stable supersalts when combined with superhalogen anions.^11,14−16^ These exceptional properties, especially when lithium clusters are doped with elements such as carbon, open the door to materials with tailored functionalities for various technological applications.^15,17,18^

Considering the potential of carbon doping to significantly modify the properties of lithium clusters, a thorough understanding of its effects is warranted. In this work, we investigate the structural and electronic properties of small neutral carbon–lithium clusters, Li_*n*–1_C (where *n* = 2, 3, ..., 10), to elucidate the influence of carbon on their physical and chemical characteristics. Using the fixed-node diffusion quantum Monte Carlo (DMC) method,^19−22^ we obtain highly accurate estimates of ground-state energy, binding energy, and dissociation energy as a function of cluster size. To explicitly account for electronic correlation effects, we also perform Hartree–Fock (HF) calculations in the complete basis set limit. Our results demonstrate that carbon doping enhances the stability of lithium clusters, with both HF interactions and electronic correlations playing a significant role. The energy required to dissociate the carbon atom from the cluster is notably higher than for lithium, highlighting the stabilizing effect of carbon. Furthermore, we compare these results with previous studies on oxygen-doped lithium clusters to highlight the relative impact of different dopants on the stability and bonding characteristics of these systems. Oxygen doping, known to affect the electronic structure and stability of lithium clusters differently than carbon, provides a valuable comparison for understanding the specific contributions of carbon doping in enhancing cluster stability. By examining these differences, we aim to isolate better the unique role of carbon doping in modifying lithium cluster properties. Overall, this study provides important insights into the properties of carbon-doped lithium clusters, contributing to a better understanding of their characteristics, which may be helpful for future research into advanced materials with specific applications.

## Computational Details

The methodology employed in this study is well-established. Therefore, we briefly summarize the key computational details. We use density functional theory (DFT)^25,26^ and quantum Monte Carlo (QMC)^27,28^ simulations to investigate the electronic properties of carbon-doped lithium clusters. The initial structure optimizations were performed using DFT with Becke’s exchange functional^29^ and the Lee–Yang–Parr (LYP) correlation functional,^30^ as implemented in the Gaussian program^23^ with the 6-311++G(3df, 3pd) basis set.

QMC calculations were then performed using the CASINO code.^31^ We first performed variational Monte Carlo (VMC) simulations, optimizing a Slater-Jastrow trial wave function.^32,33^ This wave function consists of spin-up and spin-down Slater determinants of DFT orbitals, combined with a Jastrow factor that accounts for electron–electron, electron–nucleus, and electron–nucleus–electron correlations.^34^ Diffusion Monte Carlo (DMC) was subsequently used to refine the ground-state energies, employing the fixed-node approximation.^27,28,35^ We used a time step of 0.001 au and 10.000 walkers, ensuring minimal time-step and population bias by linearly extrapolating to the zero time-step limit.^36,37^

For comparison, HF calculations were performed using the cc-pVQZ and cc-pV5Z basis sets, and results were extrapolated to the complete basis set (CBS) limit using a two-point scheme.^38^

## Results and Discussions

### Structural Analysis

**Figure 1:**
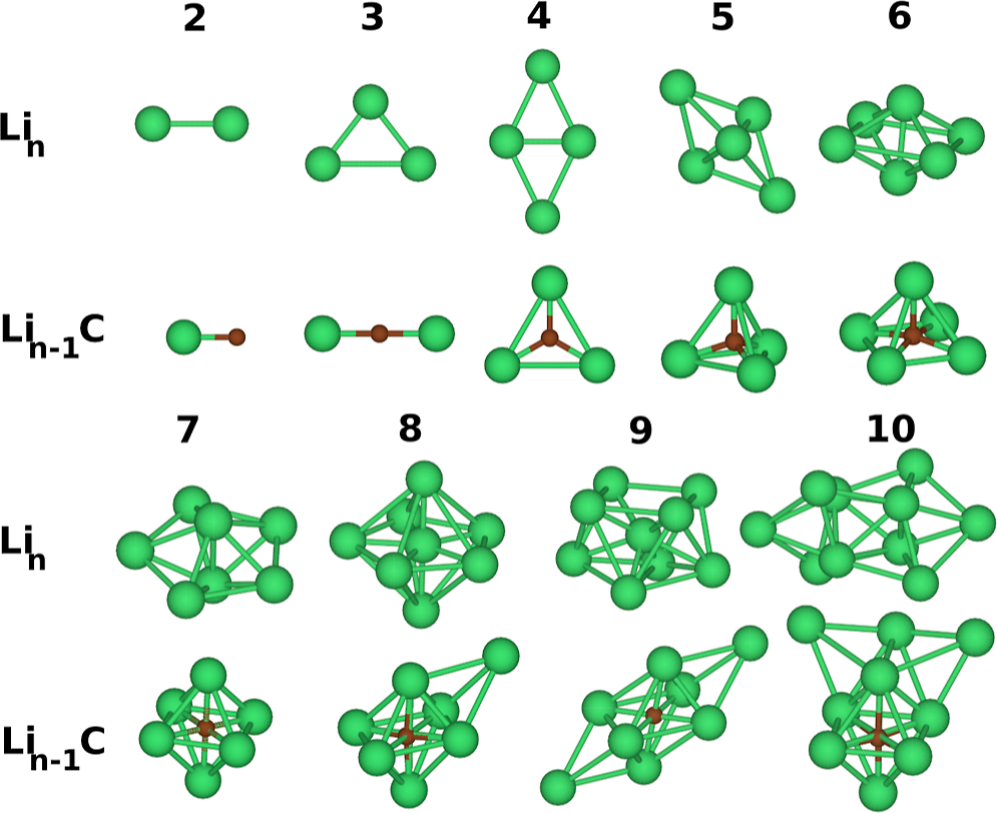
Lowest energy structures of lithium clusters (Li_*n*_) and carbon-doped lithium clusters (Li_*n*–1_C) for *n* = 2–10, as obtained from DFT/B3LYP calculations using the Gaussian03 program,^23^ based on ref (24).

To determine the atomic structure of the lithium–carbon clusters, we used a semiclassical approach based on the ABCluster code,^39^ which generated approximately 30 possible isomeric structures. We then selected the most likely geometric structures for further validation through DFT calculations, using the B3LYP functional with the 6-311++G(3df, 3pd) basis set in the Gaussian program.^23^ Additional tests with a doubled number of isomers confirmed that no novel structures emerged, indicating sufficient sampling for the systems studied. While this approach is robust for the systems investigated, we acknowledge that more extensive sampling may be required for larger or more complex heteroatomic clusters and plan to address this in future studies. To ensure the accuracy of the selected structures, we compared key parameters, such as bond lengths, with results from higher-level theoretical calculations available in the literature.^2,40,41^Figure 1 shows the set of low-lying energy structures for Li_*n*_ and Li_*n*–1_C clusters (*n* = 2, .., 10). The ground-state spin multiplicity (*M*_S_) of Li_*n*–1_C clusters is predominantly singlet or doublet for an odd and even number of atoms in the cluster, except for LiC and Li_2_C, which are quartet and triplet, respectively. While there are no available experimental equilibrium bond lengths for lithium–carbon, the selected theoretical methods have been extensively validated against high-level theoretical results for these and similar systems. This ensures the reliability of the predictions made in this work.

The atomic structures of the lowest-energy carbon-doped lithium clusters were analyzed in terms of average bond length (*d*_av_) and effective coordination number (η). Unlike the conventional coordination number (CN), η assigns different weights to bonds based on their length.^42,43^ Bonds shorter than *d*_av_ contribute with a weight greater than one, allowing η to be calculated without a bond-length cutoff. For highly symmetric structures, η equals CN. Therefore, η is more helpful in analyzing potential structural trends and has been successfully applied to study the structure of various clusters,^20,44−46^ especially metallic ones.^21,22,24,47^

**Figure 2:**
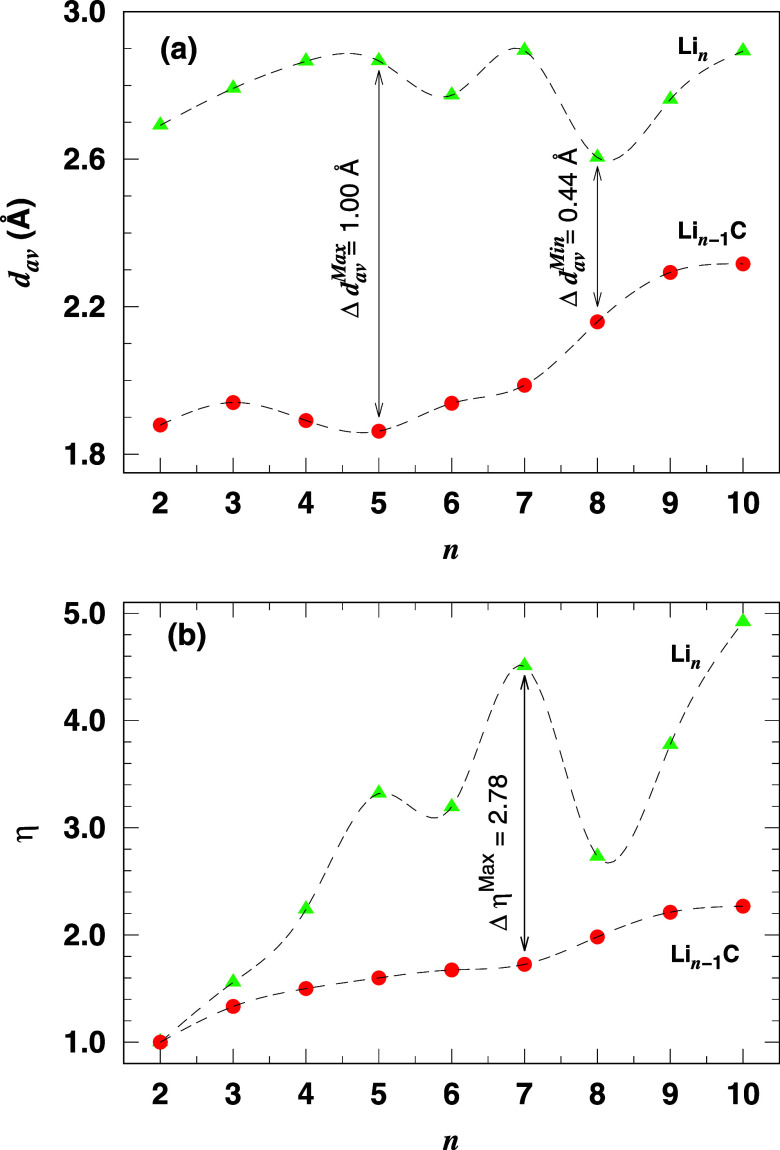
(a) The weighted average bond length (*d*_av_) and (b) the effective coordination number (η) of Li_*n*_ and Li_*n*–1_C clusters as a function of cluster size (*n*). The dashed lines are guides to the eye.

To better understand the influence of a carbon atom on the cluster structures, we plot in Figure 2a,b the average bond length (*d*_av_) and effective coordination number (η), respectively, as a function of the cluster size. In Figure 2a, it is evident that doping one carbon atom into the lithium clusters reduces the average bond length compared to the corresponding pure lithium clusters.^24^ Specifically, *d*_av_ decreases by more than 34% for *n* ≤ 6 due to the presence of the doped carbon atom. However, this effect diminishes almost monotonically with increasing cluster size, with a minimum reduction of 0.44 Å for *n* = 8. Figure 2b shows that η is sensitive enough to detect structural changes caused by substituting a lithium atom with a carbon in the clusters. For the doped clusters, η increases smoothly with increasing cluster size, while for the pure lithium clusters, it also increases but exhibits a pronounced even–odd alternation for 5 ≤ *n* ≤ 9. The substitution of lithium with carbon reduces η, indicating significant structural changes in the clusters. Notably, the cluster with *n* = 7 shows the largest variation in η in a structure where six lithium atoms surround the carbon atom.

**Figure 3:**
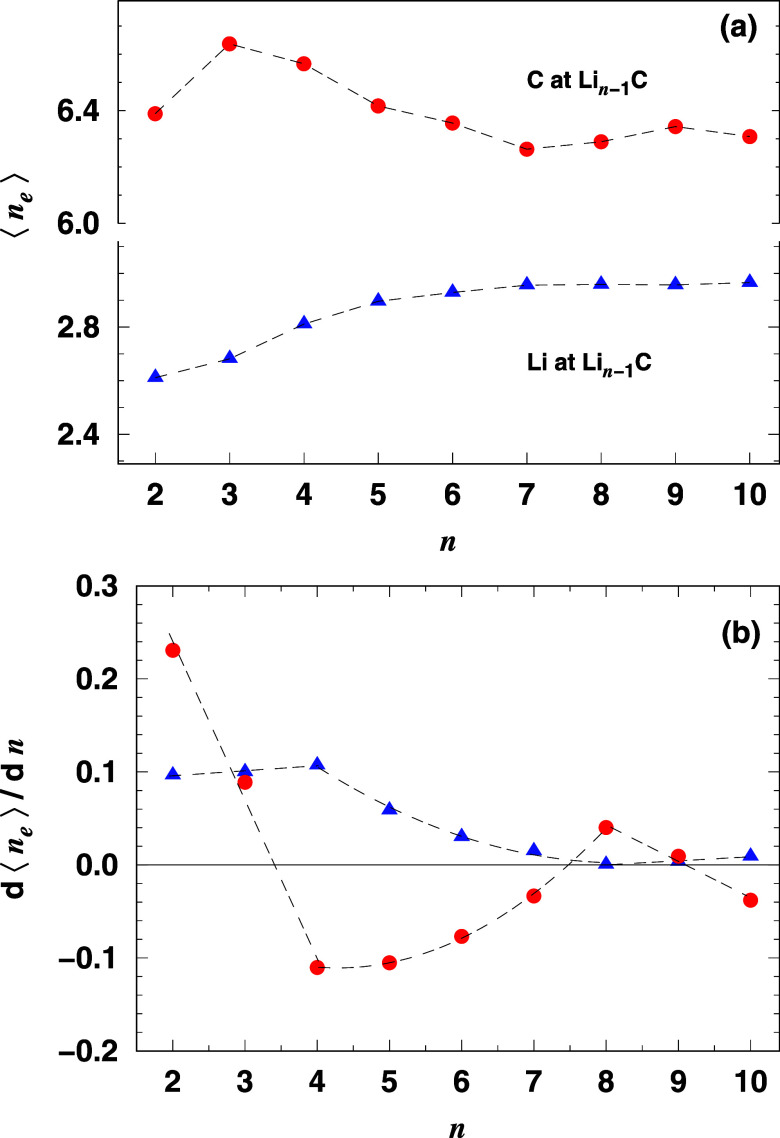
(a) The average electron population and (b) the derivative of the average electron population around atoms in Li_*n*–1_C clusters as a function of cluster size (*n*). The red circles indicate the electron population for carbon atoms, and the blue triangles for lithium atoms. The dashed lines are guides to the eye.

Next, we examine the electron population distribution around the nuclei in the studied clusters. This analysis, as shown in previous works,^21,22^ provides insights into chemical bonding and electron delocalization. The CASINO program applies the Voronoi polyhedral scheme to assess electron population around the nuclei.^31^Figure 3a shows the average electron population (⟨*n*_e_⟩) around lithium (blue triangles) and carbon (red circles) in Li_*n*–1_C clusters as a function of cluster size. For *n* ≤ 7, the electron population around lithium increases slightly, while carbon fluctuates by about 4% around 6.40. In larger clusters, populations stabilize. Substituting lithium with carbon decreases the electron population around lithium and increases it around carbon, as expected from electronegativity. In LiC, lithium loses electron population, while carbon gains 0.39. The electron population around carbon stabilizes at around 6.30 for Li_7_C, Li_8_C, and Li_9_C, and the population analysis suggests polar-covalent bonding, with carbon atoms attracting electrons and lithium atoms losing them.

Figure 3b shows the derivative of the average electron population, *d*⟨*n*_e_⟩/*dn*, which illustrates how the electron transfer develops with increasing cluster size. Positive values indicate electron accumulation, while negative values reflect electron depletion. Up to *n* = 3, both carbon and lithium atoms show an increase in electrons, with the contribution of lithium increasing slightly. However, from *n* = 4, the carbon atoms begin to lose electrons, as shown by the negative derivative values, indicating the onset of electron saturation. On the other hand, the electron donation of lithium decreases but remains positive throughout. For *n* ≥ 8, the lithium contribution stabilizes close to zero, indicating a minimal electron gain per lithium atom. The carbon derivative recovers slightly at *n* = 8 before becoming negative again at *n* = 10, reinforcing the trend toward electron saturation in carbon.

**Figure 4:**
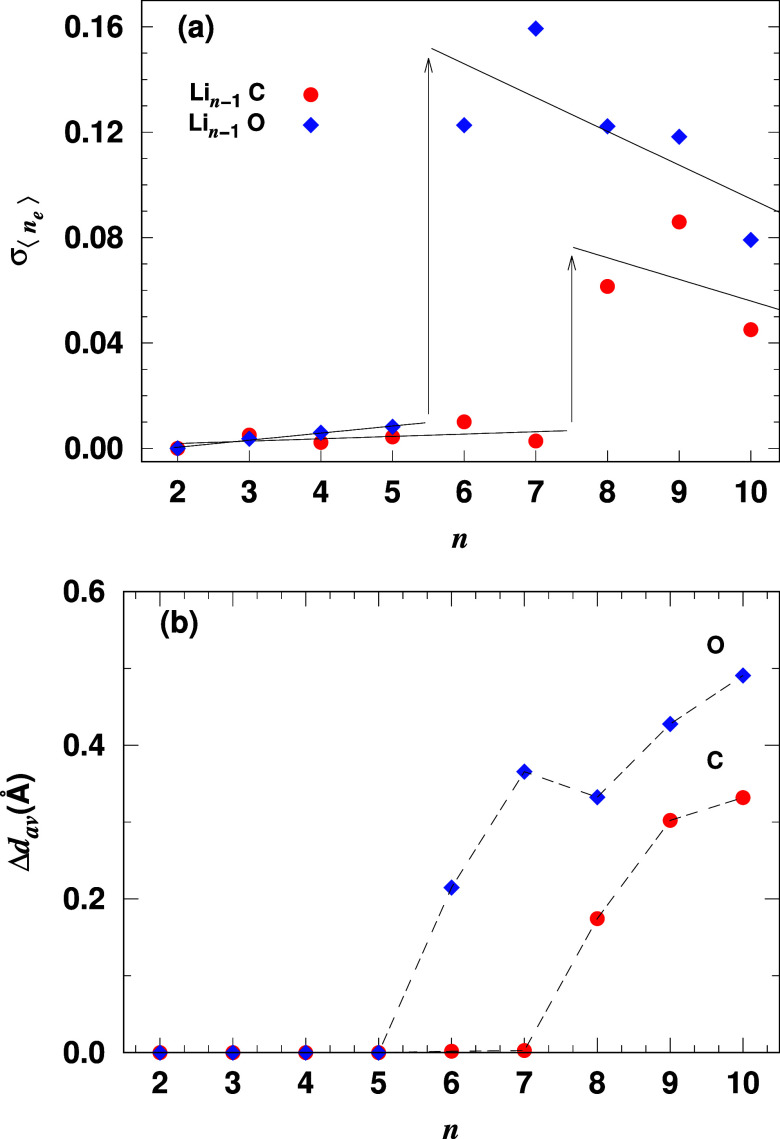
(a) Standard deviation of the electron population distribution around lithium atoms, and (b) the difference between the average bond length of the cluster and the average dopant–lithium bond length in Li_*n*–1_C and Li_*n*–1_O clusters as a function of cluster size. The arrows indicate the transition from delocalized to localized electron states. The solid lines represent a linear fit of the data, while the dashed lines are provided as guides for the eye.

Figure 4a shows the standard deviation of the electron distribution around lithium atoms , which reflects the degree of electron localization in the clusters. We compare these results with oxygen-doped lithium clusters from previous work.^22^ Initially,  increases slightly up to *n* = 5 for Li_*n*–1_O and *n* = 7 for Li_*n*–1_C, indicating a relatively even electron distribution. For larger clusters,  increases more rapidly, peaking at *n* = 7 for oxygen-doped and *n* = 9 for carbon-doped clusters. Despite the higher oxygen electronegativity, carbon-doped clusters show a slower increase in electron delocalization due to the tetravalent bonding capacity of the carbon. This slower saturation is evident in the curvature of *d*⟨*n*_e_⟩/*dn* between *n* = 4 and 8 in Figure 3b. These changes reflect structural transformations in the clusters.

Figure 4b shows the difference in average bond length between the cluster and the dopant (Δ*d*_av_ = *d*_av_ – *d*_av_^*dop*^). Δ*d*_av_ remains near zero up to *n* = 5 for oxygen and *n* = 7 for carbon, then increases sharply, indicating a shift in bonding arrangement. For *n* > 7, oxygen becomes pentacoordinate, forming a local minimum in Δ*d*_av_. When Δ*d*_av_ is near zero, lithium atoms are directly bonded to the dopant. As Δ*d*_av_ increases, electron delocalization spreads, particularly after the dopant’s charge absorption is saturated, with extra lithium atoms contributing to more delocalized electrons.

### Atomic Binding Energies

Carbon doping enhances the binding strength of the clusters. The binding energy per atom for a Li_*n*–1_C cluster is defined as^22,53^
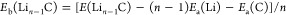
1where *E*(Li_*n*–1_C), *E*_a_(Li), and *E*_a_(C) are the total energies of the cluster, lithium atom, and carbon atom, respectively, and *n* is the total number of atoms in the cluster.

**Figure 5:**
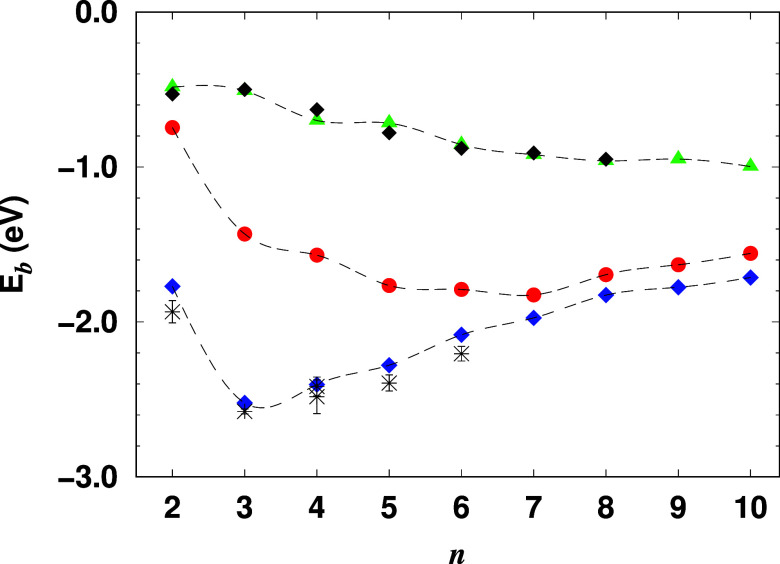
Binding energy per atom for pure lithium clusters (Li_*n*_, green triangles) and carbon-doped lithium clusters (Li_*n*–1_C, red circles) from DMC simulations (without zero-point energy correction). Theoretical data for oxygen-doped lithium clusters (blue diamonds) from ref (22) and available experimental results for pure (black diamonds) and oxygen-doped lithium clusters (black asterisks) are also included for comparison.^48−52^ All energies are in eV. Note that negative binding energy values (*E*_b_) indicate a bound system, with larger magnitudes signifying higher stability.

In Figure 5, we present the binding energies of carbon-doped lithium clusters (Li_*n*–1_C) for *n* = 2 to 10, based on DMC calculations. For comparison, pure lithium clusters (green triangles) and oxygen-doped lithium clusters (blue diamonds) are also shown, with updated results from ref (22). The statistical errors in the DMC results are smaller than the size of the symbols used in the figure. For pure lithium clusters, the binding energy (*E*_b_) decreases almost monotonically with increasing cluster size, from −0.485 ± 0.002 eV (at *n* = 2) to −0.997 ± 0.002 eV (at *n* = 10). This gradual decrease reflects a consistent reduction in binding energy as more atoms are added, indicative of a stabilization trend typical of pure metallic clusters.

In contrast, carbon-doped clusters display a more complex trend. Initially, the binding energy decreases more rapidly than in pure lithium clusters, reaching a minimum around *n* = 7. For the LiC cluster (*n* = 2), the binding energy is −0.746 ± 0.008 eV, which decreases to −1.826 ± 0.002 eV by *n* = 7, a total variation of 1.080 ± 0.008 eV. Interestingly, beyond *n* = 7, the binding energy begins to increase again, albeit at a slower rate, roughly 0.08 eV per atom. This behavior likely reflects a structural or electronic rearrangement within the cluster that increases the overall stability of larger carbon-doped clusters, suggesting a more delocalized bonding pattern as the cluster grows.

Oxygen-doped clusters, on the other hand, show a distinct minimum in binding energy much earlier, at *n* = 3. The binding energy drops sharply from −1.771 ± 0.005 eV (at *n* = 2) to −2.523 ± 0.003 eV (at *n* = 3). The doping of lithium clusters with oxygen increases the magnitude of the binding energy, thereby enhancing the stability of the doped clusters, particularly for smaller cluster sizes (*n* ≤ 3). Beyond this point, the binding energy exhibits a linear decrease in magnitude at a faster rate of approximately 0.12 eV per atom. This pronounced gain in binding energy for oxygen-doped clusters suggests that oxygen forms stronger bonds with lithium at smaller cluster sizes compared to carbon doping. The stronger bonding at *n* = 3 may be attributed to the higher electronegativity of oxygen, which enhances electrostatic interactions and results in a tightly bound cluster at smaller sizes. Beyond *n* = 3, oxygen-doped clusters stabilize in a manner similar to pure lithium clusters, though with a higher overall binding energy.

The observed minimum at *n* = 7 for carbon-doped clusters and *n* = 3 for oxygen-doped clusters could indicate different optimal electronic configurations for these dopants. In carbon-doped clusters, the rearrangement at *n* = 7 might reflect a shift in the bonding mechanism or electron delocalization that stabilizes the cluster. For oxygen-doped clusters, the early stabilization suggests that strong bonds form rapidly, but beyond *n* = 3, the system behaves similarly to pure lithium, though with slightly stronger bonding.

Overall, the DMC calculations show that the doping of lithium clusters with carbon or oxygen increases the magnitude of the binding energy, thereby enhancing the stability of the doped clusters. The improvement is more pronounced for smaller clusters, where the dopant significantly changes the electronic structure and bonding properties. However, as the size increases, the influence of the dopant decreases and the binding energies of the doped clusters approach those of the pure lithium clusters. The different trends in carbon and oxygen doping illustrate the contrasting ways in which these elements influence the stability of the cluster, with carbon causing delayed stabilization and oxygen causing early but stronger binding.

### Electronic Correlation Energy

The trends of binding energy in carbon-doped lithium clusters (Figure 5) show the general stabilization effects induced by carbon substitution. However, to fully understand the energetic mechanisms behind these trends, it is crucial to decompose the total binding energy into its HF and correlation energy components as *E*_b_ = *E*_b_^HF^ + *E*_b_^corr^ ≃ *E*_b_^DMC^. Such a decomposition provides insight into how each energy contribution varies with cluster size and how carbon doping compares to oxygen doping. These finer details reveal the underlying electronic interactions responsible for the stability observed in Figure 5. Then, the changes in HF and correlation energy contributions to the binding energy resulting from carbon doping can be, respectively, obtained as
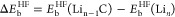
2and
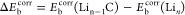
3

**Figure 6:**
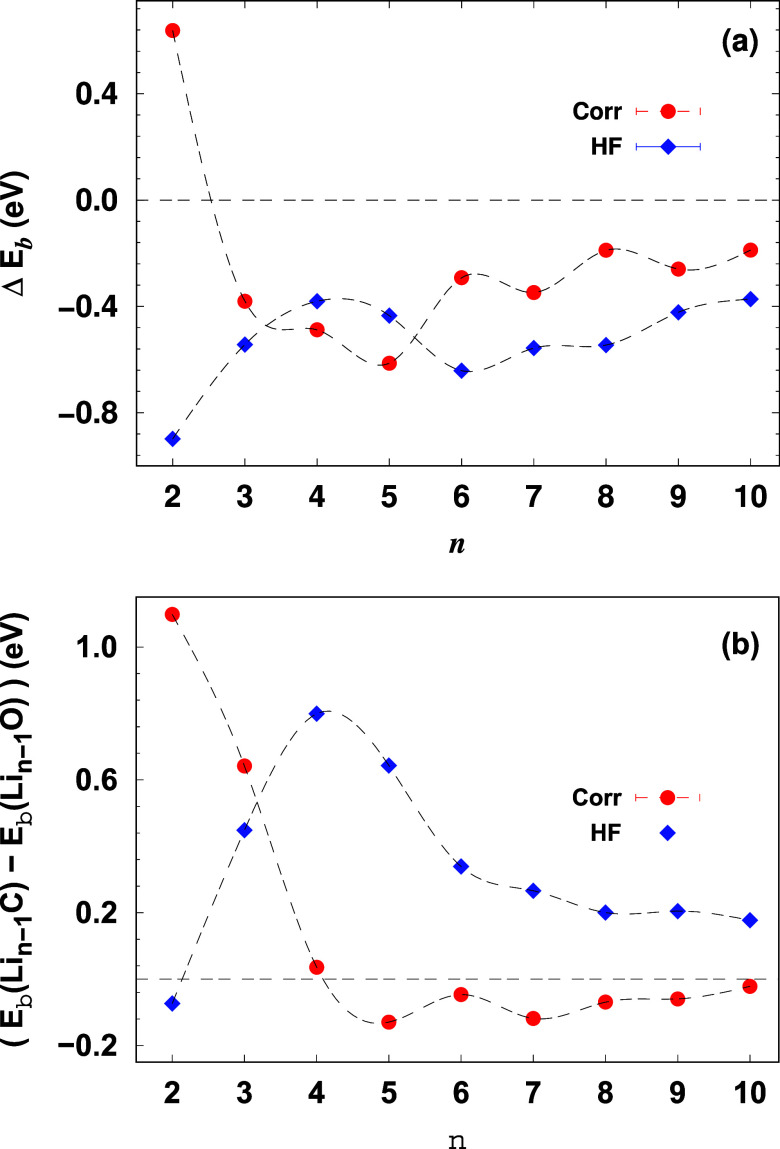
(a) Hartree–Fock (blue diamonds) and correlation (red circles) contributions to the binding energy due to carbon doping in lithium clusters. (b) The difference in Hartree–Fock and correlation contributions to the binding energies between Li_*n*–1_C and Li_*n*–1_O clusters as a function of cluster size (*n*).

In Figure 6a, blue diamonds represent the HF contribution, while red circles indicate the correlation energy contribution due to carbon doping. For *n* = 2 (LiC), the data shows a positive correlation energy gain (+0.638 ± 0.008 eV), indicating that carbon doping enhances correlation energy, compensating for the significantly reduced HF interaction (−0.899 eV), leading to overall stabilization. As cluster size increases to *n* = 3 (Li_2_C), both HF and correlation contributions decrease, with a correlation gain of −0.381 ± 0.004 eV and an HF contribution of −0.544 eV, suggesting a reduction in both energy components due to carbon doping. For *n* = 4 (Li_3_C), a reversal occurs where the correlation energy gain (−0.488 ± 0.004 eV) exceeds the HF gain (−0.381 eV), a trend that becomes more pronounced for larger clusters and differs from the behavior typically observed in oxygen-doped clusters. This trend persists in *n* = 5 (Li_4_C), where the correlation gain (−0.614 ± 0.003 eV) surpasses the HF contribution (−0.435 eV), indicating that correlation effects become increasingly significant as the cluster size grows. For *n* = 6 and beyond, an odd–even oscillation in correlation energy emerges, as seen in *n* = 6 (Li_5_C), where the correlation gain decreases in magnitude to −0.292 ± 0.003 eV while the HF contribution becomes more negative (−0.642 eV). This oscillatory behavior is characteristic of carbon doping and contrasts with the smoother energy trends observed in oxygen-doped systems.^22^

Now, we evaluate the differences in the Hartree–Fock (HF) and correlation contributions to the binding energies between carbon- and oxygen-doped lithium clusters, focusing specifically on the quantities *E*_b_^HF^(Li_n-1_C)-*E*_b_^HF^(Li_*n*–1_O) and *E*_b_^corr^(Li_*n*–1_C)-*E*_b_^corr^(Li_*n*–1_O). These differences are critical to understanding how the nature of the dopant (carbon vs oxygen) affects the balance between electrostatic interactions (captured by the HF contribution) and many-body correlation effects, which can ultimately influence the overall stability and bonding properties of the clusters.

By comparing the HF and correlation contributions for each dopant, we can determine whether the stronger binding observed in oxygen-doped clusters, especially at smaller sizes, is primarily driven by electrostatic forces or if correlation effects play a more significant role. Additionally, this comparison helps explain why carbon-doped clusters exhibit enhanced correlation effects at larger sizes, suggesting a shift in the dominant interaction mechanism as the cluster grows. As shown in Figure 6b, these differences reveal two key trends: (i) The HF contribution is generally higher in oxygen-doped clusters for most cluster sizes. This indicates that oxygen forms stronger electrostatic interactions with lithium compared to carbon. However, an exception occurs at *n* = 2, where the HF contribution in carbon-doped clusters exceeds that of oxygen-doped clusters by 0.0735 eV. (ii) For small clusters (*n* ≤ 5), the correlation contribution is larger in oxygen-doped clusters, but for larger clusters (*n* ≥ 5), the correlation contribution becomes slightly larger in carbon-doped clusters. For instance, at *n* = 5, the difference in correlation energy is 0.129 ± 0.004 eV, favoring the carbon-doped cluster. This indicates how correlation effects become more dominant in carbon-doped clusters as the system grows, in contrast to the initially stronger HF contribution in oxygen-doped clusters.

The stronger HF interaction in oxygen-doped clusters, especially for *n* ≤ 5, leads to higher binding energies in these systems compared to carbon-doped ones, due to the stronger bonding between oxygen and lithium atoms. However, for larger clusters (*n* > 5), the increased correlation effects in carbon-doped systems contribute more significantly to the binding energy. This shift in the balance between HF and correlation energy contributions indicates that carbon doping enhances many-body interactions in larger clusters, resulting in a more balanced contribution from both HF and correlation effects as the cluster size increases.

### Dissociation Energies

#### Bond Dissociation Energies and Dopant Interaction in Lithium Clusters

The interaction between the dopant atom and the host lithium atoms within the cluster depends on the cluster size. This effect can be studied by calculating the bond dissociation energy *D*_Li–X_, defined as^22^
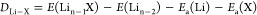
4where *E*(Li_*n*–1_X) and *E*(Li_*n*–2_) are the total energies of Li_*n*–1_X (with the dopant X = C or O) and Li_*n*–2_ clusters, respectively, and *E*_a_(X) is the total energy of a single atom X. *D*_Li–X_ represents the energy required to break the bond between the dopant and the lithium atoms in the cluster and provides insight into the bond strength.^22^

**Figure 7:**
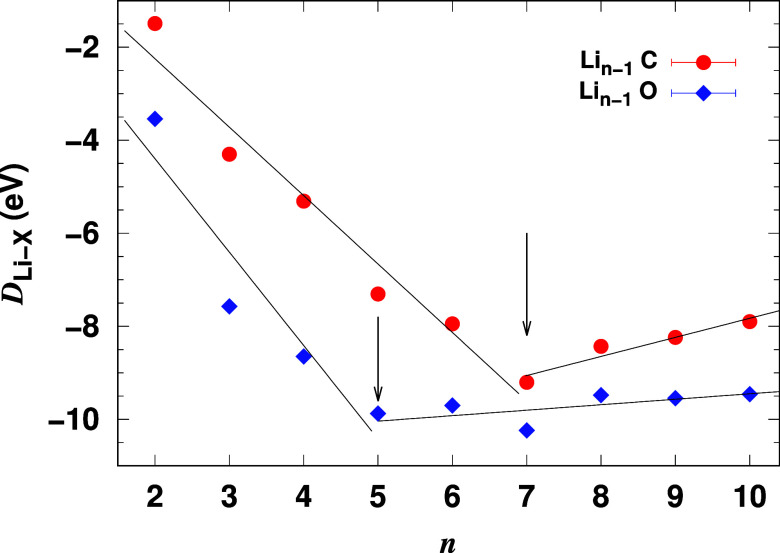
Bond dissociation energy (*D*_Li–X_) for oxygen-doped and carbon-doped lithium clusters as a function of cluster size (*n*). Arrows indicate the cluster sizes where bond saturation occurs. Scatter points represent calculated data, while solid lines show linear fits added for visual clarity.

Figure 7 shows the bond dissociation energy *D*_Li–X_ for carbon–lithium bonds (red circles) and oxygen–lithium bonds (blue diamonds) as a function of cluster size. For both dopants, *D*_Li–X_ decreases (becomes more negative) almost linearly with increasing cluster size, up to *n* = 5 for oxygen-doped clusters and *n* = 7 for carbon-doped clusters. This indicates that the bonding strength increases with cluster size up to these points. Beyond these cluster sizes, the bond strength reaches a minimum and fluctuates.

The rate of change of the bond dissociation energy, given by – *dD*_Li–X_/*dn*, or the rate of attraction  exerted by the dopant on the lithium atoms, further elucidates the nature of the bonding. For smaller cluster sizes where decreases approximately linearly,  remains nearly constant. It is estimated to be 1.47 for carbon and 2.01 for oxygen, meaning oxygen exerts a stronger attractive force on the lithium atoms, consistent with the electronegativity values of 2.55 for carbon and 3.44 for oxygen on the Pauling scale.

Although oxygen has a stronger attraction to lithium, its bond dissociation energy *D*_Li–O_ saturates at a smaller cluster size than carbon. As shown by the vertical arrows in Figure 7, the saturation occurs at *n* = 5 for oxygen-doped clusters, while for carbon, it occurs at *n* = 7. This suggests that the tetravalent nature of the carbon allows it to accommodate more lithium atoms despite its lower electronegativity. These trends provide a clearer understanding of the role of dopant type in controlling bonding interactions within lithium clusters, further supporting the conclusions drawn from Figure 4a,b. Specifically, these results demonstrate the saturation of charge absorption capacity and the evolution of electron sharing as the number of lithium atoms increases.

#### Dissociation Energies and Channels in Doped Lithium Clusters (Li_*n*–1_C and Li_*n*–1_O)

In Table 1, we present the DMC dissociation energies required to decompose a Li_*n*–1_C cluster, alongside the corresponding HF (Δ_HF_) and correlation contributions (Δ_c_). We analyze three distinct dissociation channels: Li_*n*–1_C → Li_*n*–2_C + Li, Li_*n*–1_C → Li_*n*–3_C + Li_2_ and Li_*n*–1_C → Li_*n*–1_ + C. The dissociation energies for these channels are defined as follows
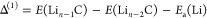
5
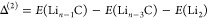
6and
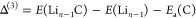
7

**Table 1:** Dissociation Energies Δ^(1)^, Δ^(2)^, and Δ^(3)^ of the Li_*n*–1_C Clusters for Three Different Dissociation Channels, as Defined by eqs 5–7, Respectively^a^

cluster	DMC dissociation energies									others	
size, *n*	Δ^(1)^	Δ_HF_^(1)^	Δ_c_^(1)^	Δ^(2)^	Δ_HF_^(2)^	Δ_c_^(2)^	Δ^(3)^	Δ_HF_^(3)^	Δ_c_^(3)^	Δ^(1)^	Δ^(2)^
2	–1.49(2)	–1.96	0.47(2)				–1.49(2)	–1.97	0.47(2)		
3	–2.81(2)	–0.31	–2.50(2)	–3.33(1)	–2.11	–1.23(1)	–3.33(1)	–2.11	–1.23(1)		
4	–1.98(1)	–0.08	–1.90(1)	–3.82(2)	–0.22	–3.59(2)	–4.75(1)	–1.71	–3.04(1)		
5	–2.55(1)	–1.01	–1.54(1)	–3.56(1)	–0.93	–2.63(1)	–6.03(1)	–2.54	–3.49(1)		
6	–1.92(1)	–2.13	0.22(1)	–3.50(1)	–2.98	–0.52(1)	–7.16(1)	–4.31	–2.85(1)		
7	–2.04(2)	–0.68	–1.36(2)	–2.99(2)	–2.64	–0.34(2)	–7.65(2)	–4.53	–3.12(2)		–2.82(11)
8	–0.78(2)	–0.70	–0.08(2)	–1.85(2)	–1.21	–0.64(2)	–7.12(2)	–4.60	–2.52(2)	–0.62	
9	–1.12(2)	–0.23	–0.89(2)	–0.93(2)	–0.75	–0.17(2)	–7.00(2)	–4.59	–2.41(2)		–0.74 – – 0.81
10	–0.90(2)	–0.18	–0.72(2)	–1.05(2)	–0.24	–0.81(2)	–7.04(1)	–3.98	–3.06(1)		

a The HF and correlation contributions to the dissociation energies are also provided. Experimental and theoretical values from the literature are included for comparison in the last two columns.^2,40,54^ Statistical errors from the DMC calculations are indicated in parentheses. All energies are reported in eV.

The dissociation energy Δ^(1)^ quantifies the energy required to remove one Li atom from the Li_*n*–1_C cluster. Notably, Δ^(1)^ exhibits an even–odd alternation with increasing *n* and becomes less negative as cluster size increases, indicating a more favorable energetics for Li removal in larger clusters. Specifically, the energy Δ^(1)^ for this removal varies from −2.81 ± 0.02 eV at *n* = 3 to −0.78 ± 0.02 eV at *n* = 8. In our analysis, we also observe that the correlation contribution Δ_c_^(1)^ is positive at *n* = 2 (for LiC) and *n* = 6 (for Li_5_C), while the HF contribution Δ_HF_^(1)^ is dominant at *n* = 2, 6, and 8. However, for other carbon-doped clusters Li_*n*–1_C with *n* = 3, 4, 7, 9 and 10, the correlation contribution is significantly larger than Δ_HF_^(1)^.

Comparatively, the dissociation channel Δ^(2)^, which involves removing a lithium dimer (Li_2_), does not always require more energy than Δ^(1)^. An exception occurs at *n* = 9 for Li_8_C, where the dimer dissociation energy Δ^(2)^ is smaller than Δ^(1)^. This behavior has also been observed in the Li_8_O cluster.^22^ Further analysis indicates that the correlation contribution Δ_c_^(2)^ predominates for *n* = 4, 5, and 10 in the dissociation channel Δ^(2)^. The experimental and theoretical data for the dissociation channels Δ^(1)^ and Δ^(2)^ are provided in the last column of Table 1, where our calculated dissociation energies demonstrate good agreement.

Finally, the energy cost of removing the carbon atom from Li_*n*–1_C clusters is given by Δ^(3)^. In comparison to the other two channels, the dissociation energy Δ^(3)^ is significantly larger for *n* > 3. Both the HF and correlation contributions are substantial, with the magnitude of correlation contribution Δ_c_^(3)^ being greater in magnitude than Δ_HF_^(3)^ for *n* = 4 and 5.

**Figure 8:**
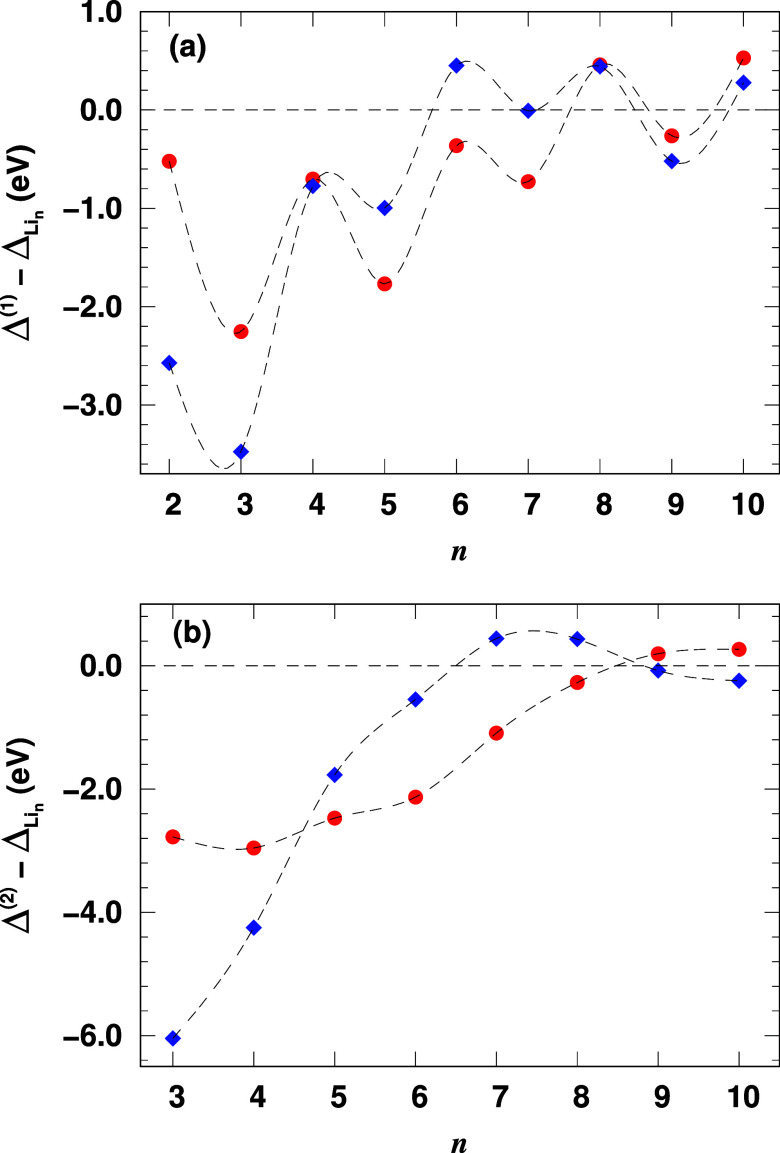
Differences in dissociation energies between doped clusters (Li_*n*–1_X) and undoped lithium clusters (Li_*n*_) of the same size for (a) dissociation of a single Li atom (channel 1) and (b) dissociation of a Li_2_ dimer (channel 2). Red circles represent X = C (Li_*n*–1_C) and blue diamonds represent X = O (Li_*n*–1_O). The dashed lines are guides to the eye.

Next, we analyze the differences in dissociation energies between carbon-doped and oxygen-doped lithium clusters compared to undoped lithium clusters of the same size, considering two different dissociation channels. Figure 8a illustrates the difference in dissociation energy, , for channel 1, which depicts the dissociation of a single Li atom, as a function of cluster size *n*. The results reveal a pronounced even–odd alternation pattern, indicating that odd-sized clusters exhibit different stability compared to even-sized clusters, for both the carbon- and oxygen-doped lithium clusters. Generally, replacing a Li atom with C or O significantly affects the energy required to remove a Li atom from smaller clusters. Oxygen-doped clusters exhibit greater thermodynamic stability against Li dissociation up to *n* = 4. For larger clusters, the stability alternates, with the carbon-doped systems becoming more stable for 5 ≥ *n* ≥ 8. Notably, undoped lithium clusters exhibit higher stability against Li dissociation compared to both doped systems for *n* = 8 and 10.

Regarding the dissociation of a Li_2_ dimer, Figure 8b shows that both carbon and oxygen doping significantly enhance the stability of small clusters. Specifically, oxygen doping lowers the dissociation energy Δ^(2)^ by 6.05 ± 0.01 eV for Li_2_O (*n* = 3) and 4.24 ± 0.02 eV for Li_3_O (*n* = 4). However, the doping effects on the dissociation energy diminish with increasing cluster size, becoming insignificant for *n* ≥ 6 in oxygen-doped clusters and for *n* ≥ 8 in carbon-doped ones. This behavior can be attributed to carbon’s greater capacity to stabilize lithium atoms in the clusters, likely due to its stronger covalent bonding interactions compared to those formed with oxygen.

## Conclusions

In this study, we employed QMC, DFT, and HF methods to investigate the structural and electronic properties of carbon-doped small lithium clusters. The lowest-energy structures were obtained using DFT, which also provided the single-particle orbitals for the VMC trial wave function. DMC calculations, based on the optimized VMC wave functions, revealed the effects of carbon doping on the atomic and electronic structure of the clusters.

Our analysis shows that carbon substitution reduces the bond length by approximately 1.00 Å and decreases the coordination number by as much as 2.78. The doped clusters exhibited a notable increase in binding energy, with enhancements in binding energy ranging from 0.26 to 1.05 eV compared to their undoped counterparts. This change is attributed to a shift in the bonding nature from purely metallic to polar covalent, driven by the presence of carbon. Additionally, the derivative of the electronic population analysis suggests that carbon saturation occurs in clusters containing six lithium atoms (Li_6_C).

The electronic correlation contributes significantly to the total binding energy, with correlation effects contributing nearly equally to the binding energy as the HF interaction in most clusters. However, for LiC, the electronic correlation slightly destabilizes the cluster, decreasing its binding energy. In terms of dissociation energy, the removal of a carbon atom requires between −7.65 ± 0.02 and −3.33 ± 0.01 eV, significantly higher than the energy required to remove a lithium atom, which ranges between −2.81 ± 0.02 and −0.78 ± 0.02 eV.

Comparing these results to oxygen-doped lithium clusters shows that carbon doping leads to stronger bonding and higher dissociation energies. Our findings provide crucial insights into the role of carbon in stabilizing lithium clusters and highlight the potential of carbon doping in the development of materials with enhanced stability and bonding properties.
